# Reevaluating carbon storage and emissions in California’s harvested wood products: implications for alternative waste parameters

**DOI:** 10.1186/s13021-026-00407-7

**Published:** 2026-01-29

**Authors:** Taylor K. Lucey, Meghan Graham MacLean, Nadia A. Tase

**Affiliations:** 1 Department of Environmental Conservation, University of Massachusetts Amherst, 160 Holdsworth Way, 01002 Amherst, MA USA; 2 California Department of Forestry and Fire Protection, Sacramento, CA 95814 USA

**Keywords:** Carbon emissions, Landfills, Waste, Paper, Harvested wood products, Climate change

## Abstract

Harvested wood and paper products can store large amounts of carbon long-term but also contribute to carbon emissions once discarded. Currently, several tools are used for inventory and reporting carbon in wood and paper products in the U.S. Carbon in wood and paper is tracked from initial manufacturing, through its lifetime, and final fate (e.g., dumps, landfills, incinerated, or recycled). Once discarded into landfills, a portion of wood and paper is assumed permanently stored; however, carbon storage of specific products can vary widely which influences carbon storage and emissions estimates. Using historical California harvest data and state-level inventory model, HWP-C vR, this research built model capacity for expanding and refining waste parameters, such as product-level decay half-lives and proportions of permanent carbon storage to reduce waste parameter uncertainty. By updating the proportion of carbon permanently stored in landfilled wood and paper products and by adding product-specific discard pathways, carbon in solid waste disposal sites cumulatively increased moderately by about 11.77 MMT CO_2_Eq and emissions decreased by about 11.18 MMT CO_2_Eq in California from 1953 to 2020. Updated parameters furthermore made it possible to compare product-level carbon storage and emissions within landfills such as newspaper, office paper, coated paper, cardboard, plywood, and lumber. The cumulative wood product categories resulted in similar amounts of carbon compared with paper products – 28.21 MMT CO_2_Eq (0.415 MMT CO_2_Eq annually) and 27.39 MMT CO_2_Eq (0.403 MMT CO_2_Eq annually) respectively; however, the carbon storage of wood products was much higher than paper, with 164.07 MMT CO_2_Eq (2.413 MMT CO_2_Eq annually) stored compared with 31.41 MMT CO_2_Eq (0.462 MMT CO_2_Eq annually) respectively. These carbon emissions and storage estimates illustrate the value in understanding carbon dynamics at the product-level particularly when considering climate impacts from landfill emissions even after product disposal.

**Supplementary Information:**

The online version contains supplementary material available at 10.1186/s13021-026-00407-7.

## Background

**Fig. 1:**
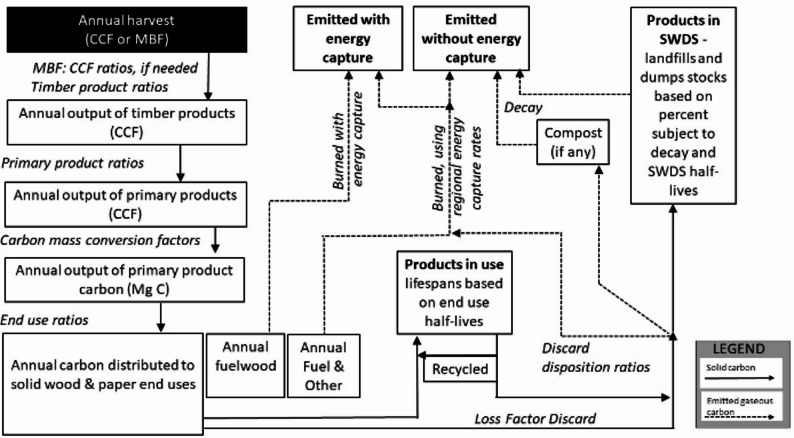
HWP-C model carbon flows schematic– reproduced with permission (K. Stockmann, pers comm, August 21, 2025)

Globally, land use and land use change account for around 21% of carbon emissions [1]. Timber production is a major component of land use, with the ability to influence carbon emissions, sequestration, and storage. The U.S. is both the largest producer and consumer of industrial roundwood which includes logs in their raw form to produce everything from lumber to pulpwood used for paper production [2]. Wood and paper products in the U.S. have been estimated to store more than 100 million metric tons of carbon dioxide equivalents (MMT CO_2_Eq) [3, 4]. However, according to U.S. EPA Waste and Materials Characterization Reports, close to one third of U.S. solid waste is composed of paper and wood products [5], stressing that the fates of these products are as important as their carbon storage potential. To inventory carbon in wood and paper products (referred to as harvested wood products, HWP), the U.S. follows the Intergovernmental Panel on Climate Change’s Tier 3 Production Approach to estimate the national HWP carbon inventory [6], which estimates carbon stored in wood and paper products from harvest to final end-use (e.g., furniture, housing, books), and disposal (e.g., burned, recycled, landfilled, Fig. 1). A small portion of HWP that are recycled return to the products in-use carbon pool, but most of the remaining HWP waste is disposed of into landfills. This approach excludes any carbon stored or emitted from HWP imported from outside of the producing region; the producing region is where trees are grown and harvested. HWP from the producing region that are exported are assumed to be used and disposed of in the same way that they would have been domestically [7]. Under the Production Approach, carbon stored in solid waste disposal sites (SWDS) such as landfills and dumps are estimated, however, emissions from these waste environments are reported separately as part of the waste sector emissions inventory [8]. Historically, only carbon from HWP is estimated following the Production Approach, and differentiating the carbon into different greenhouse gases, such as methane, is not done. Although short-lived compared to carbon dioxide, methane is a more potent greenhouse gas, and differentiating carbon emissions would impact climate forcing estimates [a^8^]. Assumptions about both HWP end-uses and discard pathways introduce room for uncertainty when estimating HWP carbon inventories [7, 9].

California has been monitoring and estimating carbon stocks in forests and harvested wood products since 2017, following the passage of Assembly Bill 1504 [10]. California’s timber harvest began to sharply decline starting in the early 1990’s and since then, forest management objectives have also shifted towards forest health restoration and fuels reduction in the region because of rapidly increasing mortality from more frequent and severe wildfires [9]. Declining harvest over the last three decades has led to a trend in the state’s HWP carbon inventory reporting in which the carbon stored in SWDS is beginning to grow more rapidly than carbon stored in products in-use. More of the wood and paper products harvested from California’s forests are being discarded than are being replaced with new products. Although this trend suggests an increase in emissions from landfilled HWP and dumps, there is also a proportion of carbon that remains inert with continued storage in solid waste environments like landfills [6, 11]. From 1952 to 2019, California reported that the estimated cumulative carbon stored in SWDS which includes landfills and dumps was 219.85 MMT CO_2_Eq while an estimated 282.70 MMT CO_2_Eq was stored in the products in-use carbon pool [9]. As more HWP are being discarded than produced from California’s forests, reducing uncertainty around HWP waste emissions, such as those associated with landfills, is becoming increasingly important for modeling and estimating the impacts of HWP carbon.

Reducing uncertainty in HWP carbon storage in- and emissions from- landfills could improve estimates of the long-term and potential future impacts of HWP carbon. One tool that has been used to estimate HWP carbon inventories is the U.S. Forest Service HWP-C v1 model and its state-level variants such as HWP-C vR [12–14]. Details about HWP-C vR including carbon pools, pathways, model functionality, and underlying assumptions have been extensively documented [13–16] and the HWP-C vR variant has been used to estimate state-level HWP carbon inventories for California, Oregon, and Washington [9, 17–19]. Default discard pathways for HWP-C vR are the same as those in the national model (USFS HWP-C v1): emitted with energy capture, emitted without energy capture, landfills, dumps, recovered/recycled, and composted (Fig. 1). Both USFS HWP-C v1 and HWP-C vR models follow the IPCC Tier 3 Production Approach [6] and use a common approach to modeling the carbon in discarded wood products, including aggregating all product end-uses into either wood or paper discard categories before estimating emissions and storage (Fig. 1). Discarded HWP carbon emissions and storage are estimated using a combination of the proportion of carbon in landfilled products that are stored permanently, and product decay rates for the remaining carbon subject to decay.

The proportion of HWP that is assumed inert in landfills is referred to as ‘fixed carbon’ in these HWP carbon models [16, 20] and is the non-degradable carbon in landfills. The default proportions in HWP-C vR and USFS HWP-C v1 [16] were originally developed from laboratory studies [21] examining the decay rates of several materials such as corrugated containers (e.g., cardboard), office paper, old newsprint, coated paper, and branches. The findings from this work were later adapted for U.S. EPA landfill decay and methane generation rates [22]. Estimated fixed carbon proportions from branches were used as a proxy for wood while fixed carbon paper categories were averaged and applied to any paper or paperboard product in USFS HWP-C v1 and HWP-C vR [16, 21]. The remaining proportion of HWP carbon in landfills is subject to decay following First-Order Decay methods and based on 2006 IPCC Tier 1 methods [23] for estimating emissions from landfills. These decay rates and fixed carbon ratios influence the estimates of the amount of greenhouse gas emissions, including carbon dioxide and methane _,_ from discarded wood products. Since the development of the underlying datasets used in USFS HWP-C v1 and its variants, more recent studies about decay rates of HWP, including differentiated paper products in landfills, have been published [11, 24, 25]. One of the more recent published updates to product-specific fixed carbon ratios and decay rates for wood and paper products is in the U.S. EPA Waste Reduction Model v-15 [26]. Each of these updates has the potential to significantly change the estimates of carbon stored and emitted from discarded wood products. Although the parameter alterations in this research primarily focus on landfills, changes to this carbon pool will influence other discard pools such as dumps as well as overall estimated emissions.

Landfill parameters are the primary focus for this research because the majority of discarded HWP are assumed to go to landfills in HWP-C vR which are a major source of greenhouse gas emissions in the US, and landfill carbon is assumed to be accumulating at a more rapid pace than in HWP still in-use in California [8, 9]. The goal of this work was to build HWP-C vR’s capacity for future updates to waste parameters such as product-level decay and fixed carbon ratios, and reduce uncertainty associated with waste parameters in HWP-C vR using California harvest data as an example. This research used the publicly available and free to download HWP-C vR model and input data for the California state-level inventory using timber harvest data from 1952 to 2019 and various other parameters necessary for HWP carbon estimation [14], with the exception of alternate end-use categories for paper products [27], and alternate fixed carbon ratios [26] to test the sensitivity of carbon storage pools and emissions from discarded HWP. Another goal of this work was to use only publicly available data for model parameters for transparency and replicability. All data management, model applications, and ratio creation were conducted in RStudio [28] unless stated otherwise.

## Methods

### Fixed carbon ratios for wood

The HWP-C vR model estimates carbon cumulatively starting from a historical year supplied by the user. Because the model follows the IPCC Production Approach [6], biogenic carbon is estimated in HWP in-use, stored in SWDS, and emitted after disposal. These carbon values are estimated as a function of the historical volume of wood products produced in a given year and do not reflect the time horizon of all emissions returning to the atmosphere which is estimated as part of the waste sector emissions [8, 14]. While the model provides emissions estimates, these emissions are officially reported in the waste sector.

We ran HWP-C vR with California timber harvest data from 1952 to 2019 using the ‘shift year’ function; “shift year” means that the previous year’s emissions are reported in the following year (i.e., initial year is 1953 and final output year is 2020). Applying alternative fixed carbon ratios to more specific HWP categories than available in HWP-C vR required both altering and expanding the existing model using R code [14] to accommodate U.S. EPA Waste Reduction Model version 15 fixed carbon ratios (Table 1) [26]. To apply more product-specific fixed carbon ratios, we needed to add more HWP discard product categories to HWP-C vR. Prior to our analysis, the 224 end use ratios in HWP-C vR were aggregated into either wood or paper products once discarded (see Table S1 for a full list of model end-uses). The proportion of landfilled products then received one of two default fixed carbon ratios depending on whether they were paper or wood (Table 1). Landfilled products and products discarded into dumps also receive one of two default half-lives for HWP subject to decay and half-lives for recovered/recycled [16, 21, 29]. Half-lives are the estimated length of time it takes for half the mass of a given material to decay. USFS HWP-C v1 and HWP-C vR assume that HWP discarded into dumps are subject to aerobic decay which means they have a shorter half-life than landfills and therefore a faster decay rate; landfills are assumed to be subject to anaerobic conditions and decay more slowly (Table 2) [16].

**Table 1:** Fixed carbon ratios for HWP in landfills in U.S. EPA WARM v-15 [26] and HWP-C vR

Product	U.S. EPA Landfills, fixed	HWP-C vR Landfills, fixed
Corrugated containers	0.55	-
Newspaper	0.84	-
Office paper	0.12	-
Coated paper	0.74	-
Dimensional lumber	0.88	-
Medium-density fiberboard	0.84	-
Wood flooring	0.95	-
Wood	-	0.77
Paper	-	0.44

To apply specific wood product fixed carbon ratios from U.S. EPA [26], we first needed to disaggregate the wood discard category in HWP-C vR. We created a crosswalk between the default discard, end-use, and primary product categories in HWP-C vR and the product category names in U.S. EPA (see Table S1 for the crosswalk) [26]. We disaggregated the ‘wood’ discard category into lumber, plywood, and a generic wood category. Primary and end-use product categories in HWP-C vR that were not explicitly identified as lumber, plywood, oriented strand board, or medium-density fiberboard remained in the default ‘wood’ discard category as a catchall for the remaining products. The default HWP-C vR fixed carbon ratio for wood (Table 2) was applied to the remaining wood products [21, 29]. About two-thirds of the end-use categories fall into the catchall ‘wood’ discard category which makes up about half of the timber harvest volume in California from 1952 to 2019.

**Table 2:** Default discard parameters in HWP-C vR

Product	Dumps (years)	Landfills, fixed (ratio)	Landfills, decay (years)	Recovered (years)
Paper	8.25	0.44	14.5	2.6
Wood	16.5	0.77	29	2.6

We applied the medium-density fiberboard fixed carbon ratio in U.S. EPA (Table 1) to oriented strand board, medium-density fiberboard, and plywood, which we labelled ‘plywood’ as an abbreviation [26]. Medium-density fiberboard and oriented strand board are more commonly made from pressurized composite, so their fixed carbon ratios and decay rates may differ from plywood fixed carbon ratios and decay rates; however, there is currently only one estimate for this type of engineered wood product (Table 2). Although U.S. EPA [26] has a fixed carbon ratio for ‘wood flooring,’ none of 224 end-use products in HWP-C vR are explicitly defined as ‘wood flooring,’ so it was excluded (Table 1). Each of the new discard categories was incorporated into the discard fate pathways which are the proportions of discarded products in dumps, landfills, recovered, composted, burned with or without energy capture (Fig. 1). Given that the discard fates are based on national estimates, we assumed that the ratio of discarded lumber and plywood would be proportionally discarded the same way as wood [16]. We then adjusted the HWP-C vR Stand Alone Model [14] to incorporate the new discard category names and applied the product specific fixed carbon ratios to run the model; however, decay rates stayed the same as default values (Table 2).

### Fixed carbon ratios for paper

**Fig. 2:**
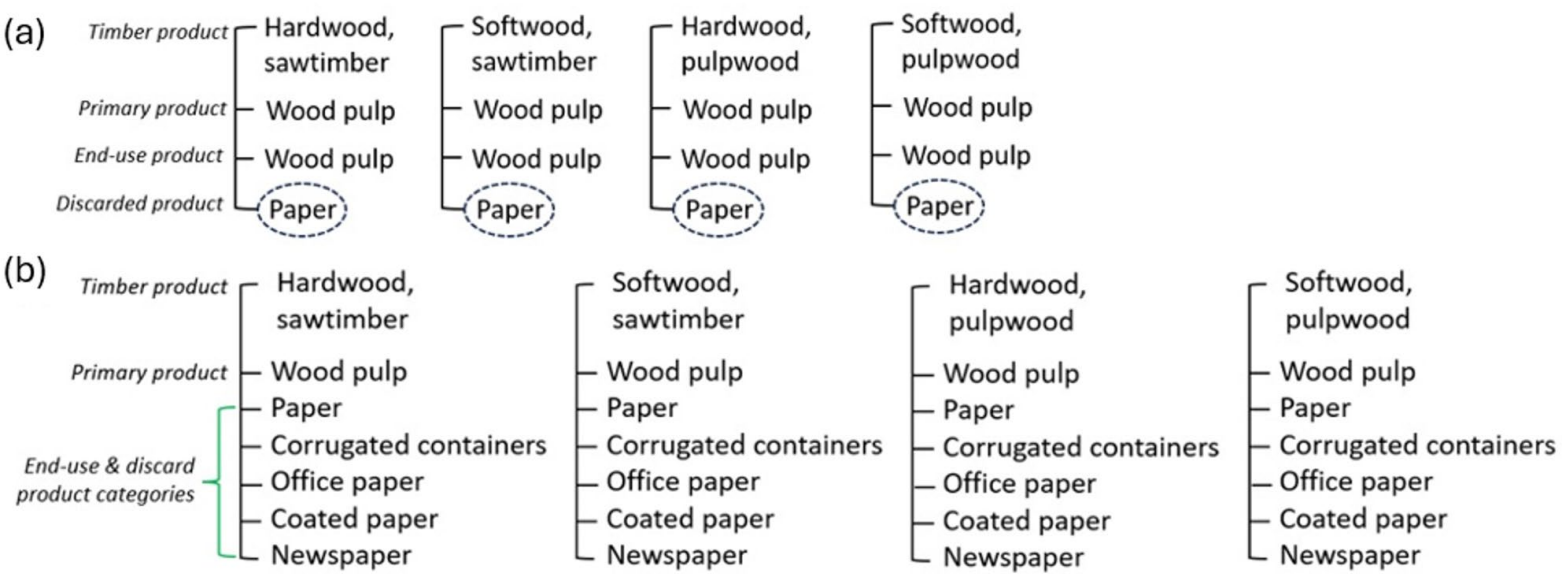
(**a**) Default HWP-C vR paper pathways, and (**b**) updated product-specific paper pathways

According to U.S. EPA [26], some paper product categories have larger fixed carbon ratios than other paper product categories, which influences the rate of emissions from solid waste disposal sites (Table 1). For example, the fixed carbon ratio for corrugated containers (i.e., cardboard) is 0.84 while the fixed carbon ratio for office paper is 0.12, suggesting that a larger proportion of office paper emissions return to the atmosphere than corrugated containers. Although it was possible to disaggregate the HWP-C vR wood products into U.S. EPA [26] lumber and plywood product categories because of existing primary and end-use products in HWP-C vR, the default variant of HWP-C vR does not have more specific end-use product categories for paper (Fig. 2); there is a single discard product category in HWP-C vR that becomes paper. Therefore, the same crosswalk methods we used for disaggregating wood in HWP-C vR to apply U.S. EPA [26] fixed carbon ratios could not be followed for specific paper products.

In HWP-C vR, a proportion of ‘wood pulp’ comes from four primary product categories– ‘hardwood, sawtimber,’ ‘softwood, sawtimber,’ ‘hardwood, pulpwood,’ ‘softwood, pulpwood’ – before becoming ‘wood pulp.’ Unless wood pulp is allocated for fuelwood (Fig. 1), the material going to each of the wood pulp end-use products remains as wood pulp in HWP-C vR until it is discarded (Fig. 2). To explore the impact that specific paper product fixed carbon ratios might have on emissions, we first needed to create new end-use product ratios to distribute wood pulp into various paper categories. We used U.S. EPA [27] waste categorization data from 1960 to 2018 to crosswalk (see additional methods and Table S2 in Supplemental Information for more details) the paper categories from U.S. EPA (Table 2) [26].

To create new end-use ratios for specific paper products, we grouped paper products in U.S. EPA’s Materials and Waste Management dataset [27] by products listed in U.S. EPA [26] and their associated half-lives. Paper products were grouped similarly to an earlier HWP carbon model, HARVCARB version 3 [30], that created waste distribution ratios based on a combination of private and public datasets, including U.S. EPA’s Materials and Waste Management dataset [27]. Some end-use products that were combined based on the same half-lives were office-type papers, books, and magazines, which were combined into the ‘office paper’ end-use and discard product categories in HWP-C vR (Table S2). Combining these paper products resulted in five paper end-use and discard categories: corrugated containers, newspaper, white/office paper, coated paper, and generic ‘other/mixed paper’ (Table 2). Like the wood product categories, any product that fell into the ‘other/mixed paper’ category received the HWP-C vR default fixed carbon ratio (Table 2).

We applied the five updated paper product categories and associated end-use ratios we created to each of the wood pulp primary product categories (Fig. 2). Applying the paper end-use ratios to wood pulp extended the end-use ratio categories from 224 to 245 products in HWP-C vR (Table S1). As with the wood, we also assumed that the ratio of specific paper categories would be discarded proportionally the same way as the generic paper category [16]. Then we applied the fixed carbon ratios for specific paper categories from U.S. EPA [26]. Like the other end-use ratios in HWP-C vR, the paper-specific ratios are based on national scale products in landfills [27].

In addition to running HWP-C vR with more product-specific fixed carbon ratios -, we also ran the model with IPCC 2019 North American default fixed carbon ratios from the IPCC Waste Model to see how a broader scale approach might influence model outputs [6]. In the IPCC Waste Model [23], the non-degradable proportion of wood is estimated to be 0.9 for wood and 0.5 for paper (bulk waste data). These estimates are close to the average fixed carbon ratios for wood and paper in U.S. EPA [26] which are 0.89 and 0.56 respectively. Default decay rates for all wood and paper products stayed the same as default values (Table 2).

### Other updates to HWP-C vR model

Although the main objective of this research was to update landfill parameters in HWP-C vR, we additionally investigated other discard pathways such as dumps. After reviewing the current defaults for the HWP-C vR dumps discard pathway, we searched for alternative, state-level discard ratios. During literature searches and communications with waste sector experts (M. Barlaz, personal communication, September 16, 2022), we found that “aerobic dump environments” are not well-defined; modern landfill depth varies , but if a dump environment is deeper than 10-12 feet, then it is likely to lead to an anaerobic decay environment. If dumps are more similar to anaerobic decay environments like landfills, this could suggest that a fixed carbon ratio analogous to those for wood and paper products in landfills could be appropriate.

We reviewed U.S. EPA [26, 31] emissions reporting and waste regulations and the Resource Conservation and Recovery Act of 1976 (RCRA) for details about modern waste environments (40 CRF § 258.1, 1997). U.S. EPA federal regulations established under RCRA banned the continued use of open dumps after 1976 and required that any open dumps cease accepting waste without a proper cover and additional infrastructure after October 9, 1994 (40 CRF § 258.1, 1997). This suggests that discard disposition ratios in HWP-C vR could be updated to reflect that around the years 1995–2000, HWP waste can be assumed to be discarded into landfills or a landfill-like environment. Given the ban on dumps, infrastructure requirements put into place under RCRA (40 CRF § 258.1, 1997), and that deeper dumps are more like an anaerobic landfill environment, we updated the HWP-C vR California input file to reflect 0% of wood and paper going to dumps after 1995 and routed any material discarded from dumps into landfills. Default HWP-C vR discard parameters only have about 2% of HWP waste being discarded into dumps after the year 1990, making this a fairly small shift in discard ratios.

## Results

We compared HWP-C vR model outputs produced with default waste parameters and model outputs produced with several parameter changes (Table 3). The parameter changes we explored in HWP-C vR only influence carbon in dumps and landfills (SWDS) and emissions without energy capture (EWOEC). No parameter changes were made that would alter California’s timber harvest volume inputs that feed the model, so the products in-use carbon pool and emissions with energy capture were unaffected by parameter changes. Products in-use carbon is affected by changes to harvest volume inputs while emissions with energy capture are estimated based on fuelwood inputs, proportions of wood waste burned for energy production onsite at manufacturing facilities, and HWP burned for energy at the end of their useful life (currently 0% in California), so landfilled emissions are excluded from this pool [14]. The five parameter changes we altered for comparison with HWP-C vR default parameters included: (1) updating paper end-use ratios [27] updating product-specific fixed carbon ratios for paper [26], (2) updating product-specific fixed carbon ratios [26] for wood, (3) altering dump discard ratios – HWP discarded into dumps after 1995 were routed to landfills, (4) applying the combined updated dump discard ratios, product-specific fixed carbon ratios for HWP, and new paper end-use ratios, and (5) applying IPCC [6] non-degradable carbon ratios for paper and wood (Table 3) for comparison with default parameters and updated parameters.

**Table 3:** Changes in SWDS and EWOEC carbon with each parameter change in HWP-C vR

	Change in pools, 2020^a^ Percent change		Cumulative total, 2020^a^ MMT CO_2_Eq		Mean annual, 1953-2020^a^ MMT CO_2_Eq	
Parameter	SWDS	EWOEC	SWDS	EWOEC	SWDS	EWOEC
Default^b^	-	-	−219.85	343.78	−3.23	5.06
Paper only^c^	−2.05	1.14	−224.36	339.86	−3.30	5.00
Wood only^c^	−2.79	1.78	−225.98	337.67	−3.32	4.97
Paper and wood^c^	−4.84	2.91	−230.49	333.76	−3.39	4.91
Dumps^d^	−0.54	0.41	−221.03	342.70	−3.25	5.04
Combined (paper, wood, dumps)^c, d^	−5.35	3.25	−231.62	332.60	−3.41	4.89
IPCC^e^	−5.65	3.61	−232.28	331.37	−3.42	4.87

^a^Negative values indicate carbon storage and positive values indicate carbon emissions. Values are rounded to the nearest hundredth

^c^U.S. EPA fixed carbon ratios for specific wood and paper products [26]

^d^HWP waste shifted from dumps to landfills in California from 1995 – 2019

^e^IPCC North American defaults for non-degradable organic carbon fraction of paper and wood [6]

After applying updated end-use ratios for paper products from U.S. EPA [27] and fixed carbon ratios for specific paper products from U.S. EPA to HWP-C vR [26], cumulative EWOEC decreased by 3.92 MMT CO_2_Eq and SWDS carbon storage increased by 4.51 MMT CO_2_Eq by the year 2020 when compared with the HWP-C vR model outputs with default parameters (Table 3). Meanwhile, applying U.S. EPA [26] fixed carbon ratios for wood resulted in 6.11 MMT CO_2_Eq decrease in EWOEC and 6.13 MMT CO_2_Eq increase in SWDS by the year 2020 when compared with default parameters (Table 3). Routing HWP waste from dumps to landfills after the year 1995 had a slight influence on the model outputs resulting in a 1.08 MMT CO_2_Eq decrease in EWOEC and 1.18 MMT CO_2_Eq increase in SWDS by the year 2020 (Table 3). The combination of U.S. EPA [26] fixed carbon ratios for wood and paper, the updated end-use ratios for specific paper categories, and updated discard pathway for dumps, led to similar cumulative changes in SWDS and EWOEC; carbon in SWDS increased by about 11.77 MMT CO_2_Eq and EWOEC decreased by about 11.18 MMT CO_2_Eq (Table 3). Applying IPCC North American defaults [6] to fixed carbon ratios for wood and paper products resulted in the greatest difference in SWDS and EWOEC compared with HWP-C vR default parameters; carbon stored in SWDS increased by about 12.43 MMT CO_2_Eq and carbon emissions from EWOEC decreased by about 12.41 MMT CO_2_Eq in 2020 (Table 3).

**Fig. 3:**
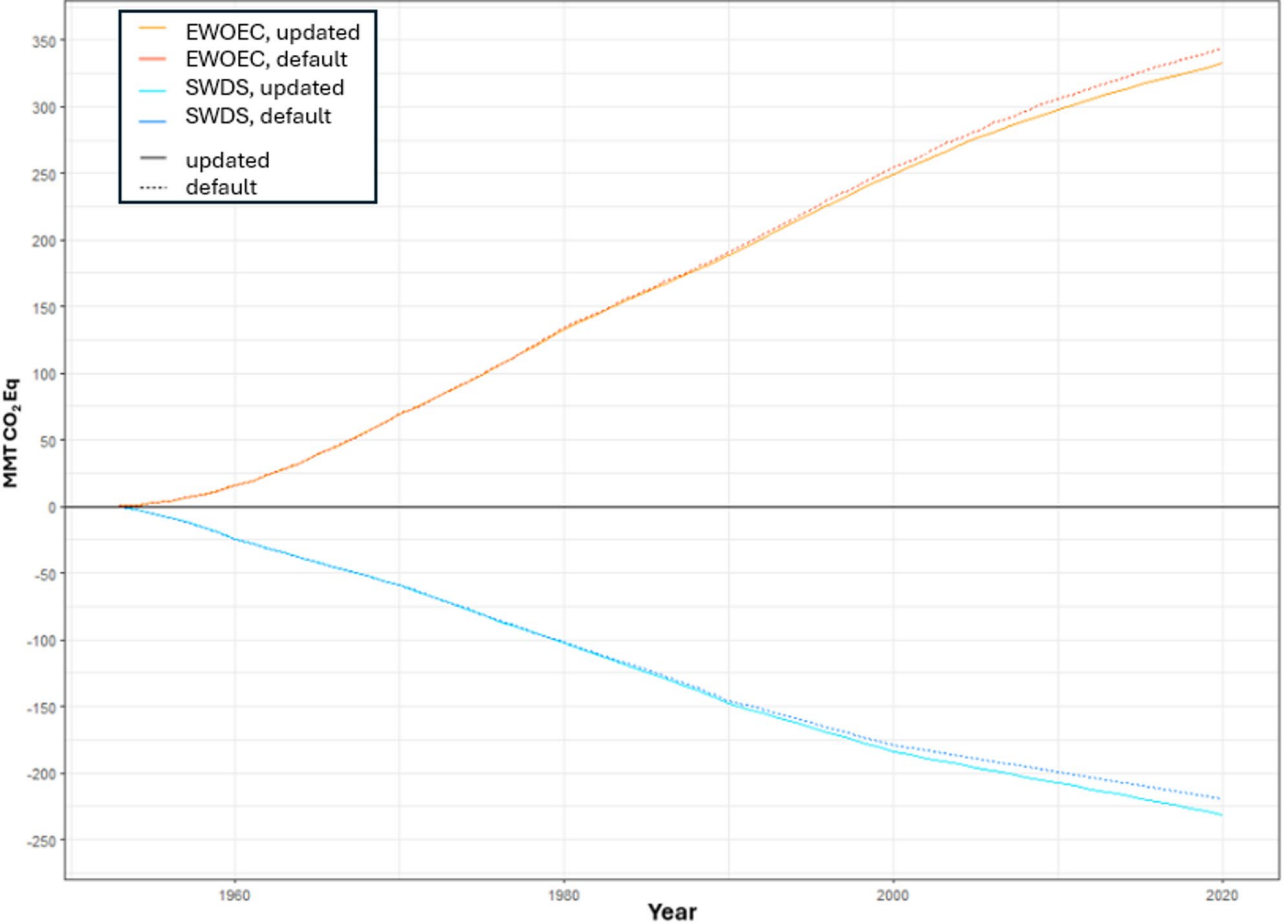
Cumulative EWOEC and SWDS in California using default and updated HWP-C vR parameters [26, 27]

**Fig. 4:**
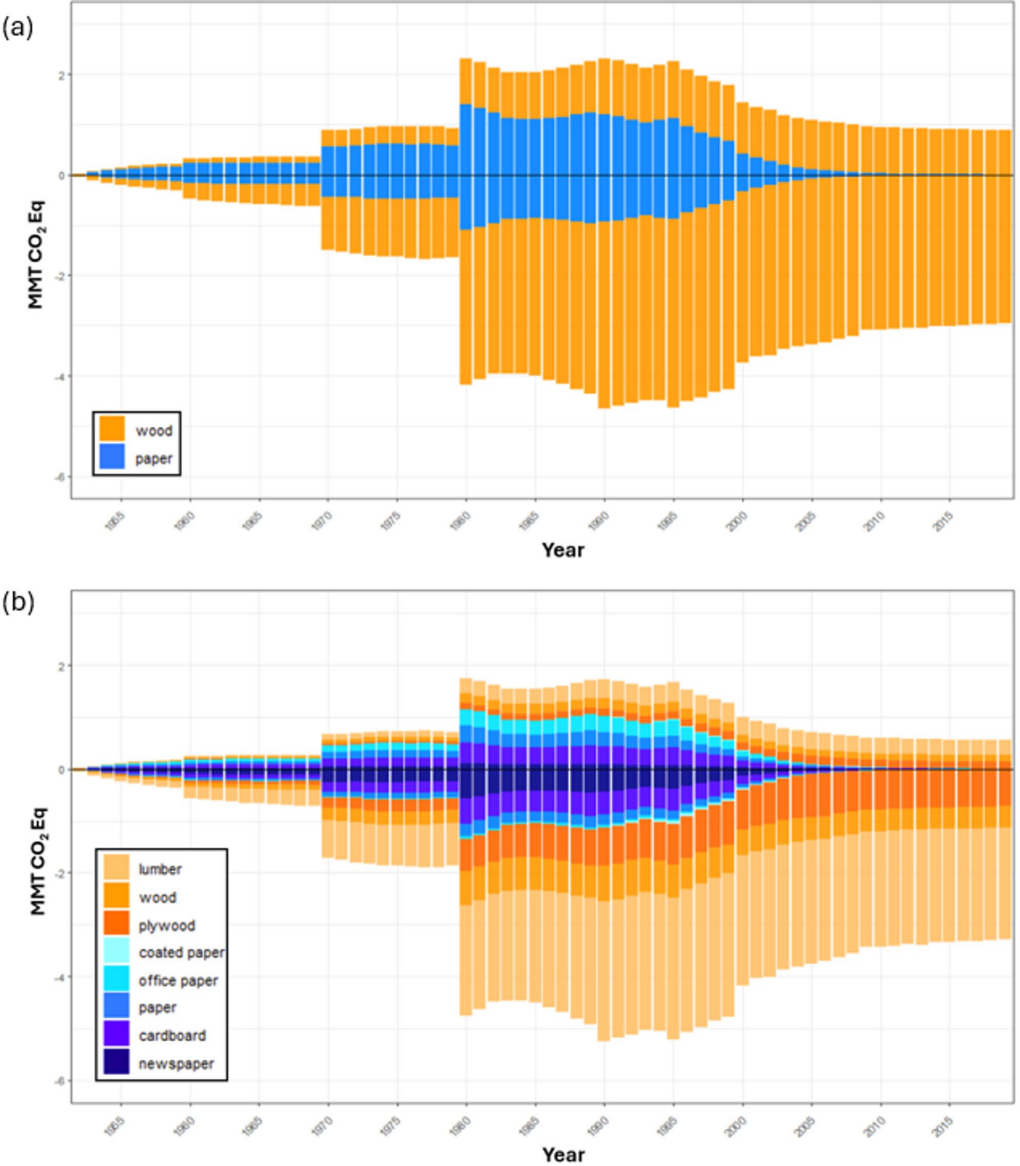
Annual landfill carbon storage and emissions (**a**) using default parameters and (**b**) updated parameters [26, 27]

Applying U.S. EPA [26] product-specific fixed carbon ratios not only increased the SWDS carbon in landfills and decreased EWOEC but also allowed for more product-specific carbon estimates, making it possible to look at the carbon impacts of the individual product categories (Fig. 4). SWDS carbon in California increased by about 4.8% primarily due to the updated fixed carbon ratios for wood products. Updated ratios for wood increased SWDS carbon by about 2.8%; an additional 2% from updated fixed carbon ratios and new end-use ratios for paper (Table 3). The decrease in EWOEC can primarily be attributed to a larger proportion of wood carbon being stored in SWDS as inert carbon, resulting in lower cumulative emissions. Emissions from wood decreased from EWOEC by about 1.7% and by about 1% from paper when compared with using the HWP-C vR default model parameters (Fig. 3). Despite the updated parameters in HWP-C vR, the cumulative carbon estimates and average annual carbon estimates in SWDS and EWOEC are similar between default HWP-C vR parameters and updated product-specific values (Fig. 3).

Because the updated parameters for HWP-C vR include product specific categories in discard pathways, it was also possible to compare emissions (represented as positive values) and storage (represented by negative values) by product type in landfills (Fig. 4). Most HWP waste resides in landfills following disposal, so we examined landfill permanent storage and waste emissions separately from the rest of the SWDS pool and associated emissions (Table 4). Estimated proportions of HWP waste going to landfills change over time, with increases in the proportion of waste going to landfills (rather than to dumps) in 1970 and 1980 (Fig. 4). Carbon stored and emitted from landfills generally mirrors timber harvest trend variation in California with some lag time between products-in-use and eventual discard [32]. Timber harvest increased until about the late 1980’s before sharply declining in the decades that followed [32]. Annual carbon storage in landfills peaked in 1990 with an estimated 5.66 MMT CO_2_Eq using the updated HWP-C vR parameters and 4.66 MMT CO_2_Eq using the default parameters (Fig. 4). Annual landfill emissions peaked in 1980 with about 1.74 MMT CO_2_Eq using updated parameters and 2.32 MMT CO_2_Eq using default parameters (Fig. 4). The peak in emissions is notably a year in which there was higher paper production, and more emissions were composed of paper waste than wood waste in 1980. The 1990 peak in carbon storage also mirrors the year with the highest wood product production in California.

When updated parameters were applied to the model, cumulative carbon stored in landfills increased by about 21.66 MMTC CO_2_Eq, increasing mean annual carbon stored in landfills by about 0.32 MMT CO_2_Eq (Table 4). Cumulative carbon in wood products and paper increased by 16.02 MMT CO_2_Eq and 5.65 MMT CO_2_Eq, respectively when compared with default parameters (Table 4). Emissions decreased by 16.02 MMT CO_2_Eq and 5.40 MMT CO_2_Eq for landfilled wood and paper products, respectively. Carbon in wood products in landfills made up most of the landfilled HWP carbon in California from 1953 to 2020, regardless of default or updated parameters (Table 4). When compared with total cumulative carbon stored in SWDS, more than 80% of the carbon stored is in wood products in landfills. Meanwhile, the cumulative landfill emissions are relatively evenly split between wood and paper products from 1953 to 2020. Changes in storage and emissions reflect not only the additional storage of individual products, but also reflect carbon diverted from dumps to landfills over time.

**Table 4:** Landfill carbon storage and emissions by product using updated fixed carbon ratios [26, 27] and default ratios

	Cumulative carbon in landfills^a^ MMT CO_2_Eq				Mean annual carbon change in landfills^a^ MMT CO_2_Eq			
	Storage		Emissions		Storage		Emissions	
Product	Updated	Default	Updated	Default	Updated	Default	Updated	Default
Wood	−25.00	−148.05	7.47	44.23	−0.37	−2.18	0.11	0.65
Paper	−5.68	−25.76	7.23	32.79	−0.08	−0.38	0.11	0.48
Lumber	−106.33	-	14.50	-	−1.56	-	0.21	-
Plywood	−32.74	-	6.24	-	−0.48	-	0.09	-
Coated paper	−0.39	-	0.14	-	−0.01	-	0.002	-
Office paper	−1.11	-	8.12	-	−0.02	-	0.12	-
Cardboard	−11.62	-	9.50	-	−0.17	-	0.14	-
Newspaper	−12.61	-	2.40	-	−0.19	-	0.04	-
*Total wood*	*−164.07*	*−148.05*	*28.21*	*44.23*	*−2.41*	*−2.18*	*−0.41*	*0.65*
*Total paper*	*−31.41*	*−25.76*	*27.39*	*32.79*	*−0.47*	*−0.38*	*−0.41*	*0.48*
*Total*	*−195.48*	*−173.82*	*55.60*	*77.02*	*−2.88*	*−2.56*	*0.82*	*1.13*

^a^MMT CO_2_Eq values are rounded to nearest hundredth, so total may vary from table. Negative values are storage estimates, and positive values are emissions estimates

## Discussion

Disaggregating and updating wood and paper discard pathways as well as fixed carbon ratios in HWP-C vR using California timber harvest data resulted in a cumulative estimated 11.18 MMT CO_2_Eq increase in carbon storage in SWDS and 12.41 MMT CO_2_Eq decrease in EWOEC from 1953 to 2020. Mean annual change estimates increased by about 0.18 MMT CO_2_Eq for SWDS and decreased by about 0.17 MMT CO_2_Eq for EWOEC. Discard half-lives and discard pathways can now be applied to more specific product types rather than the default aggregated ‘wood’ and ‘paper’ categories. This flexibility allows for a more comprehensive understanding of the impact these products have on carbon storage and emissions after they are discarded. Each parameter change we applied to HWP-C vR resulted in increased carbon storage in SWDS and decreased carbon emitted from EWOEC in California from 1953 to 2020.

When we altered the default fixed carbon ratios in HWP-C vR to match the IPCC North American defaults [6]. for wood and paper from the landfill gas model, it resulted in the biggest change to the model outputs compared with the changes resulting from using the U.S. EPA fixed carbon ratios (Table 3) [26]. This is unsurprising given that these values are meant to be applicable to wood and paper decay and carbon storage estimates for a broader region than just California and could be considered a more generalized approach. However, when applying U.S. EPA ratios [26], updated end-use ratios for paper products, as well as updating the ratio of waste going to dumps, model outputs were similar in both the SWDS pool and EWOEC; about 5% increased storage in SWDS and about 3% decreased emissions from EWOEC by 2020 (Table 3). The total cumulative changes to SWDS and EWOEC over 68 years changed slightly with the updated parameters we applied, but carbon stored in landfills increased by a much larger proportion; about 12.5% or over 20 MMT CO_2_Eq. Most of this increased storage can be attributed to the updated fixed carbon ratio for specific wood products although carbon storage from paper products did increase as well (Table 4). Digging deeper into what seems like moderately small changes to carbon in SWDS and EWOEC, it is notable just how much higher emissions are from short-lived paper products relative to their carbon storage potential. Although cumulative emissions from landfills are similar from wood and paper products, the storage component proportional to wood is significantly greater than paper products (Fig. 4).

California’s timber harvest production has been decreasing for several decades [9], so we expected that updating the fixed carbon ratios may have a muted impact on cumulative carbon storage or emissions. It is estimated that after about 2001, no primary timber harvested from California forestlands contributed to pulp [32]. Recent reports about California’s forest industry revealed that pulpwood historically was less than 2% of annual harvest volume and after 1992, any pulpwood harvested goes into “other” forest products [32]. While wood products have a higher fixed carbon ratio and influence carbon stored in SWDS, compared to paper, wood has fewer variable estimates of fixed carbon ratios. If updated parameters were applied to a state inventory with a higher volume of timber harvest or a state that produced a more diverse range of wood and paper products, it is possible that the updated fixed carbon ratios would have a greater impact on SWDS and EWOEC.

Because the fixed carbon ratios we applied to HWP-C vR come from U.S. EPA [26], they are at a national scale and could be applied to other state-level HWP carbon inventories that use the HWP-C vR model (e.g., Oregon, Washington and Colorado). While we also used national waste characterization studies from U.S. EPA [27] to create the end-use ratios for specific paper products, individual states could seek to use their own state-level waste characterization studies. We investigated the waste characterization studies created by California Department of Resources Recycling and Recovery (CalRecycle) [33] to see if they could be used to create new end-use ratios for paper products, but they did not have as much historical data as U.S. EPA, which dates to 1960 [27]. For more recent analyses of waste, CalRecycle ratios could be used; however, more research is needed to disaggregate the product categories in CalRecycle because product specific categories differ from U.S. EPA [26, 27].

An additional finding from this research was that the discard category of ‘dumps’ in HWP-C vR is unlikely applicable after about the mid-1990’s or the year 2000 (M. Barlaz, personal communication, September 16, 2022). Aerobic decay implies a more rapid decay rate than a landfill environment. For example, HWP-C vR currently assumes a half-life of 8.25 years and 16.5 years for paper and wood, respectively, when they are discarded into dumps [16]. These half-lives are almost twice as fast as half-lives for products discarded into landfills. Shifting HWP waste from dumps to landfills after 1995 resulted in the smallest change compared with other parameters from the default parameters, with about a 0.5% increase in SWDS and a 0.3% decrease in EWOEC (Table 3). A caveat for the discard category of ‘dumps’ is that it is likely that the half-lives for HWP in dumps are more similar to landfill environments, so they may also decay at a similar rate and warrant a closer look at the default HWP-C vR dumps decay rates (Table 2). However, without further evidence or current data sources, we did not update half-lives for HWP in dumps; future research could examine the discard category of dumps more closely.

Another caveat in this research is that using waste characterization studies to create paper end-use ratios, regardless of whether they are at a national or state level, is that these studies include imported and domestic HWP waste, and the IPCC Production Approach excludes imported HWP carbon. Timber harvest volume inputs (timber harvest from California’s forests) for HWP-C vR are not affected by fixed carbon ratio changes and therefore the Production Approach is still being followed; however, the ratios themselves are more representative of U.S. HWP consumption rather than production estimates. Although we searched for paper industry production data and published reports on historical paper production, much of this data was inconsistently collected or not publicly accessible. Currently, there is no consistent, publicly available paper production data that could be used to create these types of end-use categories for California. If data like this were to become available, the model is now flexible enough to include new paper end-use categories with the parameter changes we have made.

The volume of imported HWP originating from outside of the U.S. can readily be estimated because the port of entry is always recorded, it is more challenging to track HWP flows between U.S. states [34] and even more challenging to disaggregate imported and domestically produced HWP once they are discarded. If the ratio of pulp produced in California going to various paper products is different than what is being discarded nationally and reported in U.S. EPA [27] waste studies, then this could have some influence on storage or emissions from HWP. However, as previously mentioned, the volume of paper production from primary harvest in California was very low and then stopped in the 2000’s, so the impact on emissions and storage will be minimal if the IPCC Production Approach is followed. Wood and paper waste comprise almost a third of U.S. municipal solid waste, which means they are contributing to a substantial proportion of total landfills emissions. And while these emissions are reported under waste sector greenhouse gas reporting, one key to mitigating climate change is mapping and finetuning the many pathways with which these emissions originate.

## Conclusion

This work updated the state-level variant of the USFS HWP-C v1 model, HWP-C vR, using California timber harvest data from 1952 to 2019 to include more product-specific categories in waste streams, increasing the model’s flexibility. Although we used California timber harvest data, other states could use publicly available timber harvest data such as Timber Product Output surveys [35] to use HWP-V vR. We relied on publicly available data such as U.S. EPA [26] and national waste characterization studies [27] to update model parameters, so that this work could be applied to other states or to the national inventory. Users can now manually alter and update discard pathways, half-lives, and fixed carbon ratios by specific product types. Future work could further refine the disaggregation of the generic ‘wood’ category when empirical data is available for additional products. For example, there are structural differences between engineered products that currently have the same fixed carbon ratio as plywood, but these product-level differences likely impact decay rates, fixed carbon ratios, and therefore may impact carbon emissions or storage. Future work could additionally update product specific discard half-lives by altering the HWP-C vR input file and running the Stand Alone Model Code with the additional model flexibility from this work [14].

As more empirical data are collected over time, there will be more opportunities to improve current carbon inventory models that will help inform climate change mitigation strategies. It is important to investigate the underlying assumptions that determine the estimated fate of HWP carbon and emissions. Even in a state like California that has declining timber harvest and very little paper production (from pulpwood harvested in California), we found that by updating the fixed carbon ratios of HWP to be more product-specific, there was a moderate increase in carbon storage in solid waste environments by about 5% and a decrease in emissions without energy capture by about 3% which equates to approximately − 11.77 MMT CO_2_Eq and 11.18 MMTC CO_2_Eq, respectively. Our hope for this work is that it will be replicated in other states to update and refine HWP carbon inventory and emissions estimates to reduce uncertainty.

## Supplementary Information

Supplementary Material 1.

## Data Availability

The latest version of the California variant and other state variants of HWP-C vR are publicly available at [https://jeremygroom.github.io/HWP-vR-Documentation/] with relevant documentation and input files, including California harvest data. As addition public fork from Jeremygroom/HWP-C-vR: mainpage, [https://github.com/tklucey/HWP-C-vR-discard-parameters/tree/main], has been added to GitHub which contains updated model parameters and input files used in this research.

## Abbreviations

CalRecycle: California Department of Resources Recycling and Recovery
EWOEC: Emissions without energy capture
HWP: Harvested wood product
IPCC: Intergovernmental Panel on Climate Change
MMT CO_2_Eq: Million metric tons of carbon dioxide equivalent
RCRA: Resource Conservation and Recovery Act
SWDS: Solid waste disposal site
U.S. EPA WARM: US Environmental Protection Agency Waste Reduction Model
