# Supplementary material for: Reevaluating carbon storage and emissions in California’s harvested wood products: implications for alternative waste parameters
Source: Carbon Balance Manag. 2026 Jan 29;21:43. doi: 10.1186/s13021-026-00407-7 (PMC12924229; doi:10.1186/s13021-026-00407-7)
Supplement: Supplementary file 1 — Supplementary Material 1. [file 13021_2026_407_MOESM1_ESM.docx]

**Supplemental Information**

Additional Methods –

To create new end use product categories for paper in HWP-C vR, we used publicly available waste the U.S. EPA Key Facts and Figures data, Sustainable Materials Management – Materials and Waste Management in data: *Table 18. Products Generated with Detail on Nondurables in the U.S. Municipal Waste* and *Table 22. Products Generated with Detail on Containers and Packaging in the U.S. Municipal Waste Stream,* 1960 – 2018 (in tons) [[29](#USEPA2020SMM)]. Prior to 2010, data was collected either on 10-year or 5-year intervals, so we applied those ratios to the year data was collected as well as following years until another data collection year occurred.

Product categories were aggregated based on paper product half-lives in landfills according to U.S. EPA [[25](#USEPA2020),[29](#USEPA2020SMM)]. Newspaper values are listed as “Products - Nondurable goods – Newspapers/Mechanical Papers” and were combined with “directories” [[29](#USEPA2020SMM)]. Coated paper is listed as “Products – Nondurable goods – Other paper nondurable goods – magazines” and combined with “marketing mail” [[29](#USEPA2020SMM)]. Corrugated containers (i.e., cardboard) are listed as “Products – Containers and Packaging – Paper & Paperboard Pkg – Corrugated Boxes.” Office paper was listed as “Products - Nondurable goods – Other paper nondurable goods – office-type papers” and combined with “books,” “books and magazines” [[29](#USEPA2020SMM)]. The “other paper” category is the remaining proportion of paper waste (products that did not explicitly fit in the listed four product categories) and received the original, default fixed carbon ratio in HWP-C vR [[14](#Groom2025)]. These products also include “Products – Nondurable goods – Other paper nondurable goods –” such as “other commercial printing,” “tissue paper and towels,” “paper plates and cups,” and “other nonpackaging paper” ” [[29](#USEPA2020SMM)]. The paper waste categories in Table S2 have been added to an updated input file for use in HWP-C vR with product-specific fixed carbon ratios from U.S. EPA.^25^ Although CalRecycle [[33](#CalRecycle2022)] has intermittent waste stream data that also includes many of these paper product types, data collection did not begin until the year 1999. U.S. EPA^28^ has been collecting waste stream data going back to 1960 which provides more historical ratios of paper waste for HWP-C vR. HWP-C vR users could use CalRecycle [[33](#CalRecycle2022)] waste stream data for California wood products with similar caveats as using U.S. EPA waste data – they are sample estimates from all waste, and they include imported wood and paper products and therefore would be outside of the IPCC Production Approach boundaries for state inventory reporting.

**Table S1.** Full crosswalk of updated end-use product categories in HWP-C vR and new “Discard Product” category.

| **Timber**  **Product ID** | **Primary**  **Product ID** | **End Use**  **ID** | **Discard**  **ID** | **Timber**  **Product** | **Primary**  **Product** | **End Use**  **Product** | **Discard**  **Product** |
| --- | --- | --- | --- | --- | --- | --- | --- |
| 1 | 1 | 1 | 1 | hardwood, sawtimber | fuelwood and other | fuelwood and other | fuelwood |
| 2 | 8 | 48 | 1 | softwood, sawtimber | fuelwood and other | fuelwood and other | fuelwood |
| 3 | 15 | 95 | 1 | hardwood, pulpwood | fuelwood and other | fuelwood and other | fuelwood |
| 4 | 22 | 142 | 1 | softwood, pulpwood | fuelwood and other | fuelwood and other | fuelwood |
| 1 | 2 | 2 | 2 | hardwood, sawtimber | lumber | manufacturing, other manufacturing | lumber |
| 1 | 2 | 3 | 2 | hardwood, sawtimber | lumber | rail and railcar, n/a | lumber |
| 1 | 2 | 4 | 2 | hardwood, sawtimber | lumber | packaging and shipping, n/a | lumber |
| 1 | 2 | 5 | 2 | hardwood, sawtimber | lumber | manufacturing, furniture | lumber |
| 1 | 2 | 6 | 2 | hardwood, sawtimber | lumber | other, n/a | lumber |
| 1 | 2 | 7 | 2 | hardwood, sawtimber | lumber | new nonresidential, other | lumber |
| 1 | 2 | 8 | 2 | hardwood, sawtimber | lumber | new nonresidential, new nonres buildings | lumber |
| 1 | 2 | 9 | 2 | hardwood, sawtimber | lumber | residential r and r, n/a | lumber |
| 1 | 2 | 10 | 2 | hardwood, sawtimber | lumber | new housing, manufactured housing | lumber |
| 1 | 2 | 11 | 2 | hardwood, sawtimber | lumber | new housing, single family | lumber |
| 1 | 2 | 12 | 2 | hardwood, sawtimber | lumber | new housing, multifamily | lumber |
| 2 | 9 | 49 | 2 | softwood, sawtimber | lumber | residential r and r, n/a | lumber |
| 2 | 9 | 50 | 2 | softwood, sawtimber | lumber | packaging and shipping, n/a | lumber |
| 2 | 9 | 51 | 2 | softwood, sawtimber | lumber | manufacturing, other manufacturing | lumber |
| 2 | 9 | 52 | 2 | softwood, sawtimber | lumber | manufacturing, furniture | lumber |
| 2 | 9 | 53 | 2 | softwood, sawtimber | lumber | rail and railcar, n/a | lumber |
| 2 | 9 | 54 | 2 | softwood, sawtimber | lumber | new nonresidential, new nonres buildings | lumber |
| 2 | 9 | 55 | 2 | softwood, sawtimber | lumber | other, n/a | lumber |
| 2 | 9 | 56 | 2 | softwood, sawtimber | lumber | new housing, multifamily | lumber |
| 2 | 9 | 57 | 2 | softwood, sawtimber | lumber | new housing, manufactured housing | lumber |
| 2 | 9 | 58 | 2 | softwood, sawtimber | lumber | new housing, single family | lumber |
| 2 | 9 | 59 | 2 | softwood, sawtimber | lumber | new nonresidential, other | lumber |
| 3 | 16 | 96 | 2 | hardwood, pulpwood | lumber | rail and railcar, n/a | lumber |
| 3 | 16 | 97 | 2 | hardwood, pulpwood | lumber | packaging and shipping, n/a | lumber |
| 3 | 16 | 98 | 2 | hardwood, pulpwood | lumber | other, n/a | lumber |
| 3 | 16 | 99 | 2 | hardwood, pulpwood | lumber | manufacturing, furniture | lumber |
| 3 | 16 | 100 | 2 | hardwood, pulpwood | lumber | new housing, multifamily | lumber |
| 3 | 16 | 101 | 2 | hardwood, pulpwood | lumber | new nonresidential, other | lumber |
| 3 | 16 | 102 | 2 | hardwood, pulpwood | lumber | new housing, single family | lumber |
| 3 | 16 | 103 | 2 | hardwood, pulpwood | lumber | new nonresidential, new nonres buildings | lumber |
| 3 | 16 | 104 | 2 | hardwood, pulpwood | lumber | new housing, manufactured housing | lumber |
| 3 | 16 | 105 | 2 | hardwood, pulpwood | lumber | residential r and r, n/a | lumber |
| 3 | 16 | 106 | 2 | hardwood, pulpwood | lumber | manufacturing, other manufacturing | lumber |
| 4 | 23 | 143 | 2 | softwood, pulpwood | lumber | residential r and r, n/a | lumber |
| 4 | 23 | 144 | 2 | softwood, pulpwood | lumber | manufacturing, furniture | lumber |
| 4 | 23 | 145 | 2 | softwood, pulpwood | lumber | new housing, manufactured housing | lumber |
| 4 | 23 | 146 | 2 | softwood, pulpwood | lumber | new housing, multifamily | lumber |
| 4 | 23 | 147 | 2 | softwood, pulpwood | lumber | new nonresidential, new nonres buildings | lumber |
| 4 | 23 | 148 | 2 | softwood, pulpwood | lumber | new nonresidential, other | lumber |
| 4 | 23 | 149 | 2 | softwood, pulpwood | lumber | manufacturing, other manufacturing | lumber |
| 4 | 23 | 150 | 2 | softwood, pulpwood | lumber | packaging and shipping, n/a | lumber |
| 4 | 23 | 151 | 2 | softwood, pulpwood | lumber | other, n/a | lumber |
| 4 | 23 | 152 | 2 | softwood, pulpwood | lumber | new housing, single family | lumber |
| 4 | 23 | 153 | 2 | softwood, pulpwood | lumber | rail and railcar, n/a | lumber |
| 1 | 3 | 13 | 3 | hardwood, sawtimber | non-structural panels | manufacturing, other manufacturing | plywood |
| 1 | 3 | 14 | 3 | hardwood, sawtimber | non-structural panels | new housing, multifamily | plywood |
| 1 | 3 | 15 | 3 | hardwood, sawtimber | non-structural panels | new housing, single family | plywood |
| 1 | 3 | 16 | 3 | hardwood, sawtimber | non-structural panels | residential r and r, n/a | plywood |
| 1 | 3 | 17 | 3 | hardwood, sawtimber | non-structural panels | new nonresidential, new nonres buildings | plywood |
| 1 | 3 | 18 | 3 | hardwood, sawtimber | non-structural panels | new nonresidential, other | plywood |
| 1 | 3 | 19 | 3 | hardwood, sawtimber | non-structural panels | rail and railcar, n/a | plywood |
| 1 | 3 | 20 | 3 | hardwood, sawtimber | non-structural panels | manufacturing, furniture | plywood |
| 1 | 3 | 21 | 3 | hardwood, sawtimber | non-structural panels | new housing, manufactured housing | plywood |
| 1 | 3 | 22 | 3 | hardwood, sawtimber | non-structural panels | packaging and shipping, n/a | plywood |
| 1 | 3 | 23 | 3 | hardwood, sawtimber | non-structural panels | other, n/a | plywood |
| 1 | 4 | 24 | 3 | hardwood, sawtimber | oriented strandboard (OSB) | new housing, multifamily | plywood |
| 1 | 4 | 25 | 3 | hardwood, sawtimber | oriented strandboard (OSB) | rail and railcar, n/a | plywood |
| 1 | 4 | 26 | 3 | hardwood, sawtimber | oriented strandboard (OSB) | new housing, single family | plywood |
| 1 | 4 | 27 | 3 | hardwood, sawtimber | oriented strandboard (OSB) | new housing, manufactured housing | plywood |
| 1 | 4 | 28 | 3 | hardwood, sawtimber | oriented strandboard (OSB) | manufacturing, furniture | plywood |
| 1 | 4 | 29 | 3 | hardwood, sawtimber | oriented strandboard (OSB) | new nonresidential, new nonres buildings | plywood |
| 1 | 4 | 30 | 3 | hardwood, sawtimber | oriented strandboard (OSB) | manufacturing, other manufacturing | plywood |
| 1 | 4 | 31 | 3 | hardwood, sawtimber | oriented strandboard (OSB) | packaging and shipping, n/a | plywood |
| 1 | 4 | 32 | 3 | hardwood, sawtimber | oriented strandboard (OSB) | other, n/a | plywood |
| 1 | 4 | 33 | 3 | hardwood, sawtimber | oriented strandboard (OSB) | residential r and r, n/a | plywood |
| 1 | 4 | 34 | 3 | hardwood, sawtimber | oriented strandboard (OSB) | new nonresidential, other | plywood |
| 1 | 6 | 36 | 3 | hardwood, sawtimber | plywood | new housing, manufactured housing | plywood |
| 1 | 6 | 37 | 3 | hardwood, sawtimber | plywood | new housing, multifamily | plywood |
| 1 | 6 | 38 | 3 | hardwood, sawtimber | plywood | residential r and r, n/a | plywood |
| 1 | 6 | 39 | 3 | hardwood, sawtimber | plywood | new nonresidential, new nonres buildings | plywood |
| 1 | 6 | 40 | 3 | hardwood, sawtimber | plywood | new nonresidential, other | plywood |
| 1 | 6 | 41 | 3 | hardwood, sawtimber | plywood | rail and railcar, n/a | plywood |
| 1 | 6 | 42 | 3 | hardwood, sawtimber | plywood | manufacturing, furniture | plywood |
| 1 | 6 | 43 | 3 | hardwood, sawtimber | plywood | manufacturing, other manufacturing | plywood |
| 1 | 6 | 44 | 3 | hardwood, sawtimber | plywood | packaging and shipping, n/a | plywood |
| 1 | 6 | 45 | 3 | hardwood, sawtimber | plywood | other, n/a | plywood |
| 1 | 6 | 46 | 3 | hardwood, sawtimber | plywood | new housing, single family | plywood |
| 2 | 10 | 60 | 3 | softwood, sawtimber | non-structural panels | manufacturing, other manufacturing | plywood |
| 2 | 10 | 61 | 3 | softwood, sawtimber | non-structural panels | other, n/a | plywood |
| 2 | 10 | 62 | 3 | softwood, sawtimber | non-structural panels | new housing, single family | plywood |
| 2 | 10 | 63 | 3 | softwood, sawtimber | non-structural panels | rail and railcar, n/a | plywood |
| 2 | 10 | 64 | 3 | softwood, sawtimber | non-structural panels | packaging and shipping, n/a | plywood |
| 2 | 10 | 65 | 3 | softwood, sawtimber | non-structural panels | new housing, manufactured housing | plywood |
| 2 | 10 | 66 | 3 | softwood, sawtimber | non-structural panels | residential r and r, n/a | plywood |
| 2 | 10 | 67 | 3 | softwood, sawtimber | non-structural panels | new nonresidential, other | plywood |
| 2 | 10 | 68 | 3 | softwood, sawtimber | non-structural panels | manufacturing, furniture | plywood |
| 2 | 10 | 69 | 3 | softwood, sawtimber | non-structural panels | new housing, multifamily | plywood |
| 2 | 10 | 70 | 3 | softwood, sawtimber | non-structural panels | new nonresidential, new nonres buildings | plywood |
| 2 | 11 | 71 | 3 | softwood, sawtimber | oriented strandboard (OSB) | rail and railcar, n/a | plywood |
| 2 | 11 | 72 | 3 | softwood, sawtimber | oriented strandboard (OSB) | new nonresidential, other | plywood |
| 2 | 11 | 73 | 3 | softwood, sawtimber | oriented strandboard (OSB) | new housing, manufactured housing | plywood |
| 2 | 11 | 74 | 3 | softwood, sawtimber | oriented strandboard (OSB) | residential r and r, n/a | plywood |
| 2 | 11 | 75 | 3 | softwood, sawtimber | oriented strandboard (OSB) | new nonresidential, new nonres buildings | plywood |
| 2 | 11 | 76 | 3 | softwood, sawtimber | oriented strandboard (OSB) | other, n/a | plywood |
| 2 | 11 | 77 | 3 | softwood, sawtimber | oriented strandboard (OSB) | packaging and shipping, n/a | plywood |
| 2 | 11 | 78 | 3 | softwood, sawtimber | oriented strandboard (OSB) | new housing, multifamily | plywood |
| 2 | 11 | 79 | 3 | softwood, sawtimber | oriented strandboard (OSB) | new housing, single family | plywood |
| 2 | 11 | 80 | 3 | softwood, sawtimber | oriented strandboard (OSB) | manufacturing, other manufacturing | plywood |
| 2 | 11 | 81 | 3 | softwood, sawtimber | oriented strandboard (OSB) | manufacturing, furniture | plywood |
| 2 | 13 | 83 | 3 | softwood, sawtimber | plywood | residential r and r, n/a | plywood |
| 2 | 13 | 84 | 3 | softwood, sawtimber | plywood | manufacturing, furniture | plywood |
| 2 | 13 | 85 | 3 | softwood, sawtimber | plywood | new housing, single family | plywood |
| 2 | 13 | 86 | 3 | softwood, sawtimber | plywood | new housing, multifamily | plywood |
| 2 | 13 | 87 | 3 | softwood, sawtimber | plywood | manufacturing, other manufacturing | plywood |
| 2 | 13 | 88 | 3 | softwood, sawtimber | plywood | other, n/a | plywood |
| 2 | 13 | 89 | 3 | softwood, sawtimber | plywood | rail and railcar, n/a | plywood |
| 2 | 13 | 90 | 3 | softwood, sawtimber | plywood | new nonresidential, new nonres buildings | plywood |
| 2 | 13 | 91 | 3 | softwood, sawtimber | plywood | new housing, manufactured housing | plywood |
| 2 | 13 | 92 | 3 | softwood, sawtimber | plywood | packaging and shipping, n/a | plywood |
| 2 | 13 | 93 | 3 | softwood, sawtimber | plywood | new nonresidential, other | plywood |
| 3 | 17 | 107 | 3 | hardwood, pulpwood | non-structural panels | manufacturing, other manufacturing | plywood |
| 3 | 17 | 108 | 3 | hardwood, pulpwood | non-structural panels | new housing, multifamily | plywood |
| 3 | 17 | 109 | 3 | hardwood, pulpwood | non-structural panels | other, n/a | plywood |
| 3 | 17 | 110 | 3 | hardwood, pulpwood | non-structural panels | residential r and r, n/a | plywood |
| 3 | 17 | 111 | 3 | hardwood, pulpwood | non-structural panels | new nonresidential, new nonres buildings | plywood |
| 3 | 17 | 112 | 3 | hardwood, pulpwood | non-structural panels | packaging and shipping, n/a | plywood |
| 3 | 17 | 113 | 3 | hardwood, pulpwood | non-structural panels | new nonresidential, other | plywood |
| 3 | 17 | 114 | 3 | hardwood, pulpwood | non-structural panels | new housing, single family | plywood |
| 3 | 17 | 115 | 3 | hardwood, pulpwood | non-structural panels | new housing, manufactured housing | plywood |
| 3 | 17 | 116 | 3 | hardwood, pulpwood | non-structural panels | manufacturing, furniture | plywood |
| 3 | 17 | 117 | 3 | hardwood, pulpwood | non-structural panels | rail and railcar, n/a | plywood |
| 3 | 18 | 118 | 3 | hardwood, pulpwood | oriented strandboard (OSB) | manufacturing, other manufacturing | plywood |
| 3 | 18 | 119 | 3 | hardwood, pulpwood | oriented strandboard (OSB) | packaging and shipping, n/a | plywood |
| 3 | 18 | 120 | 3 | hardwood, pulpwood | oriented strandboard (OSB) | other, n/a | plywood |
| 3 | 18 | 121 | 3 | hardwood, pulpwood | oriented strandboard (OSB) | manufacturing, furniture | plywood |
| 3 | 18 | 122 | 3 | hardwood, pulpwood | oriented strandboard (OSB) | rail and railcar, n/a | plywood |
| 3 | 18 | 123 | 3 | hardwood, pulpwood | oriented strandboard (OSB) | new nonresidential, new nonres buildings | plywood |
| 3 | 18 | 124 | 3 | hardwood, pulpwood | oriented strandboard (OSB) | new housing, single family | plywood |
| 3 | 18 | 125 | 3 | hardwood, pulpwood | oriented strandboard (OSB) | new housing, manufactured housing | plywood |
| 3 | 18 | 126 | 3 | hardwood, pulpwood | oriented strandboard (OSB) | residential r and r, n/a | plywood |
| 3 | 18 | 127 | 3 | hardwood, pulpwood | oriented strandboard (OSB) | new nonresidential, other | plywood |
| 3 | 18 | 128 | 3 | hardwood, pulpwood | oriented strandboard (OSB) | new housing, multifamily | plywood |
| 3 | 20 | 130 | 3 | hardwood, pulpwood | plywood | residential r and r, n/a | plywood |
| 3 | 20 | 131 | 3 | hardwood, pulpwood | plywood | packaging and shipping, n/a | plywood |
| 3 | 20 | 132 | 3 | hardwood, pulpwood | plywood | new housing, manufactured housing | plywood |
| 3 | 20 | 133 | 3 | hardwood, pulpwood | plywood | new housing, single family | plywood |
| 3 | 20 | 134 | 3 | hardwood, pulpwood | plywood | new housing, multifamily | plywood |
| 3 | 20 | 135 | 3 | hardwood, pulpwood | plywood | other, n/a | plywood |
| 3 | 20 | 136 | 3 | hardwood, pulpwood | plywood | manufacturing, other manufacturing | plywood |
| 3 | 20 | 137 | 3 | hardwood, pulpwood | plywood | rail and railcar, n/a | plywood |
| 3 | 20 | 138 | 3 | hardwood, pulpwood | plywood | new nonresidential, new nonres buildings | plywood |
| 3 | 20 | 139 | 3 | hardwood, pulpwood | plywood | manufacturing, furniture | plywood |
| 3 | 20 | 140 | 3 | hardwood, pulpwood | plywood | new nonresidential, other | plywood |
| 4 | 24 | 154 | 3 | softwood, pulpwood | non-structural panels | new housing, single family | plywood |
| 4 | 24 | 155 | 3 | softwood, pulpwood | non-structural panels | manufacturing, furniture | plywood |
| 4 | 24 | 156 | 3 | softwood, pulpwood | non-structural panels | other, n/a | plywood |
| 4 | 24 | 157 | 3 | softwood, pulpwood | non-structural panels | packaging and shipping, n/a | plywood |
| 4 | 24 | 158 | 3 | softwood, pulpwood | non-structural panels | new nonresidential, new nonres buildings | plywood |
| 4 | 24 | 159 | 3 | softwood, pulpwood | non-structural panels | manufacturing, other manufacturing | plywood |
| 4 | 24 | 160 | 3 | softwood, pulpwood | non-structural panels | new nonresidential, other | plywood |
| 4 | 24 | 161 | 3 | softwood, pulpwood | non-structural panels | residential r and r, n/a | plywood |
| 4 | 24 | 162 | 3 | softwood, pulpwood | non-structural panels | new housing, multifamily | plywood |
| 4 | 24 | 163 | 3 | softwood, pulpwood | non-structural panels | rail and railcar, n/a | plywood |
| 4 | 24 | 164 | 3 | softwood, pulpwood | non-structural panels | new housing, manufactured housing | plywood |
| 4 | 25 | 165 | 3 | softwood, pulpwood | oriented strandboard (OSB) | manufacturing, furniture | plywood |
| 4 | 25 | 166 | 3 | softwood, pulpwood | oriented strandboard (OSB) | manufacturing, other manufacturing | plywood |
| 4 | 25 | 167 | 3 | softwood, pulpwood | oriented strandboard (OSB) | new nonresidential, other | plywood |
| 4 | 25 | 168 | 3 | softwood, pulpwood | oriented strandboard (OSB) | new housing, single family | plywood |
| 4 | 25 | 169 | 3 | softwood, pulpwood | oriented strandboard (OSB) | new housing, multifamily | plywood |
| 4 | 25 | 170 | 3 | softwood, pulpwood | oriented strandboard (OSB) | new housing, manufactured housing | plywood |
| 4 | 25 | 171 | 3 | softwood, pulpwood | oriented strandboard (OSB) | residential r and r, n/a | plywood |
| 4 | 25 | 172 | 3 | softwood, pulpwood | oriented strandboard (OSB) | rail and railcar, n/a | plywood |
| 4 | 25 | 173 | 3 | softwood, pulpwood | oriented strandboard (OSB) | packaging and shipping, n/a | plywood |
| 4 | 25 | 174 | 3 | softwood, pulpwood | oriented strandboard (OSB) | other, n/a | plywood |
| 4 | 25 | 175 | 3 | softwood, pulpwood | oriented strandboard (OSB) | new nonresidential, new nonres buildings | plywood |
| 4 | 27 | 177 | 3 | softwood, pulpwood | plywood | rail and railcar, n/a | plywood |
| 4 | 27 | 178 | 3 | softwood, pulpwood | plywood | new nonresidential, other | plywood |
| 4 | 27 | 179 | 3 | softwood, pulpwood | plywood | other, n/a | plywood |
| 4 | 27 | 180 | 3 | softwood, pulpwood | plywood | manufacturing, other manufacturing | plywood |
| 4 | 27 | 181 | 3 | softwood, pulpwood | plywood | new nonresidential, new nonres buildings | plywood |
| 4 | 27 | 182 | 3 | softwood, pulpwood | plywood | packaging and shipping, n/a | plywood |
| 4 | 27 | 183 | 3 | softwood, pulpwood | plywood | new housing, manufactured housing | plywood |
| 4 | 27 | 184 | 3 | softwood, pulpwood | plywood | new housing, multifamily | plywood |
| 4 | 27 | 185 | 3 | softwood, pulpwood | plywood | new housing, single family | plywood |
| 4 | 27 | 186 | 3 | softwood, pulpwood | plywood | manufacturing, furniture | plywood |
| 4 | 27 | 187 | 3 | softwood, pulpwood | plywood | residential r and r, n/a | plywood |
| 1 | 5 | 35 | 4 | hardwood, sawtimber | other industrial products | other industrial products | wood |
| 2 | 12 | 82 | 4 | softwood, sawtimber | other industrial products | other industrial products | wood |
| 3 | 19 | 129 | 4 | hardwood, pulpwood | other industrial products | other industrial products | wood |
| 4 | 26 | 176 | 4 | softwood, pulpwood | other industrial products | other industrial products | wood |
| 5 | 29 | 189 | 4 | hardwood, poles | hardwood, poles | hardwood, poles | wood |
| 6 | 30 | 190 | 4 | softwood, poles | softwood, poles | softwood, poles | wood |
| 7 | 31 | 191 | 4 | hardwood, pilings | hardwood, pilings | hardwood, pilings | wood |
| 8 | 32 | 192 | 4 | softwood, pilings | softwood, pilings | softwood, pilings | wood |
| 9 | 33 | 193 | 4 | hardwood, mine props | hardwood, mine props | hardwood, mine props | wood |
| 10 | 34 | 194 | 4 | softwood, mine props | softwood, mine props | softwood, mine props | wood |
| 11 | 35 | 195 | 4 | hardwood, posts | hardwood, posts | hardwood, posts | wood |
| 12 | 36 | 196 | 4 | softwood, posts | softwood, posts | softwood, posts | wood |
| 13 | 37 | 197 | 4 | hardwood, fuelwood | hardwood, fuelwood | hardwood, fuelwood | wood |
| 14 | 38 | 198 | 4 | softwood, fuelwood | softwood, fuelwood | softwood, fuelwood | wood |
| 15 | 39 | 199 | 4 | hardwood, non-sawtimber | hardwood, non-sawtimber | hardwood, non-sawtimber | wood |
| 16 | 40 | 200 | 4 | softwood, non-sawtimber | softwood, non-sawtimber | softwood, non-sawtimber | wood |
| 17 | 41 | 201 | 4 | hardwood, ties | hardwood, ties | hardwood, ties | wood |
| 18 | 42 | 202 | 4 | softwood, ties | softwood, ties | softwood, ties | wood |
| 19 | 43 | 203 | 4 | hardwood, coop bolts | hardwood, coop bolts | hardwood, coop bolts | wood |
| 20 | 44 | 204 | 4 | softwood, coop bolts | softwood, coop bolts | softwood, coop bolts | wood |
| 21 | 45 | 205 | 4 | hardwood, acid/dist. | hardwood, acid/dist. | hardwood, acid/dist. | wood |
| 22 | 46 | 206 | 4 | softwood, acid/dist. | softwood, acid/dist. | softwood, acid/dist. | wood |
| 23 | 47 | 207 | 4 | hardwood, float logs | hardwood, float logs | hardwood, float logs | wood |
| 24 | 48 | 208 | 4 | softwood, float logs | softwood, float logs | softwood, float logs | wood |
| 25 | 49 | 209 | 4 | hardwood, trap float | hardwood, trap float | hardwood, trap float | wood |
| 26 | 50 | 210 | 4 | softwood, trap float | softwood, trap float | softwood, trap float | wood |
| 27 | 51 | 211 | 4 | hardwood, misc-conv. | hardwood, misc-conv. | hardwood, misc-conv. | wood |
| 28 | 52 | 212 | 4 | softwood, misc-conv. | softwood, misc-conv. | softwood, misc-conv. | wood |
| 29 | 53 | 213 | 4 | hardwood, nav stores | hardwood, nav stores | hardwood, nav stores | wood |
| 30 | 54 | 214 | 4 | softwood, nav stores | softwood, nav stores | softwood, nav stores | wood |
| 31 | 55 | 215 | 4 | hardwood, cull logs | hardwood, cull logs | hardwood, cull logs | wood |
| 32 | 56 | 216 | 4 | softwood, cull logs | softwood, cull logs | softwood, cull logs | wood |
| 33 | 57 | 217 | 4 | hardwood, sm rnd wd | hardwood, sm rnd wd | hardwood, sm rnd wd | wood |
| 34 | 58 | 218 | 4 | softwood, sm rnd wd | softwood, sm rnd wd | softwood, sm rnd wd | wood |
| 35 | 59 | 219 | 4 | hardwood, grn bio cv | hardwood, grn bio cv | hardwood, grn bio cv | wood |
| 36 | 60 | 220 | 4 | softwood, grn bio cv | softwood, grn bio cv | softwood, grn bio cv | wood |
| 37 | 61 | 221 | 4 | hardwood, dry bio cv | hardwood, dry bio cv | hardwood, dry bio cv | wood |
| 38 | 62 | 222 | 4 | softwood, dry bio cv | softwood, dry bio cv | softwood, dry bio cv | wood |
| 39 | 63 | 223 | 4 | hardwood, sp wood pr | hardwood, sp wood pr | hardwood, sp wood pr | wood |
| 40 | 64 | 224 | 4 | softwood, sp wood pr | softwood, sp wood pr | softwood, sp wood pr | wood |
| 1 | 7 | 47 | 5 | hardwood, sawtimber | wood pulp | newspaper | newspaper |
| 2 | 14 | 94 | 5 | softwood, sawtimber | wood pulp | newspaper | newspaper |
| 3 | 21 | 141 | 5 | hardwood, pulpwood | wood pulp | newspaper | newspaper |
| 4 | 28 | 188 | 5 | softwood, pulpwood | wood pulp | newspaper | newspaper |
| 4 | 24 | 237 | 5 | softwood, pulpwood | wood pulp | newspaper | newspaper |
| 1 | 7 | 225 | 6 | hardwood, sawtimber | wood pulp | coated paper | coated paper |
| 2 | 14 | 229 | 6 | softwood, sawtimber | wood pulp | coated paper | coated paper |
| 3 | 21 | 233 | 6 | hardwood, pulpwood | wood pulp | coated paper | coated paper |
| 4 | 24 | 238 | 6 | softwood, pulpwood | wood pulp | coated paper | coated paper |
| 4 | 28 | 242 | 6 | softwood, pulpwood | wood pulp | coated paper | coated paper |
| 1 | 7 | 226 | 7 | hardwood, sawtimber | wood pulp | corrugated container | corrugated container |
| 2 | 14 | 230 | 7 | softwood, sawtimber | wood pulp | corrugated container | corrugated container |
| 3 | 21 | 234 | 7 | hardwood, pulpwood | wood pulp | corrugated container | corrugated container |
| 4 | 24 | 239 | 7 | softwood, pulpwood | wood pulp | corrugated container | corrugated container |
| 4 | 28 | 243 | 7 | softwood, pulpwood | wood pulp | corrugated container | corrugated container |
| 1 | 7 | 227 | 8 | hardwood, sawtimber | wood pulp | paper | paper |
| 2 | 14 | 231 | 8 | softwood, sawtimber | wood pulp | paper | paper |
| 3 | 21 | 235 | 8 | hardwood, pulpwood | wood pulp | paper | paper |
| 4 | 24 | 240 | 8 | softwood, pulpwood | wood pulp | paper | paper |
| 4 | 28 | 244 | 8 | softwood, pulpwood | wood pulp | paper | paper |
| 1 | 7 | 228 | 9 | hardwood, sawtimber | wood pulp | office paper | office paper |
| 2 | 14 | 232 | 9 | softwood, sawtimber | wood pulp | office paper | office paper |
| 3 | 21 | 236 | 9 | hardwood, pulpwood | wood pulp | office paper | office paper |
| 4 | 24 | 241 | 9 | softwood, pulpwood | wood pulp | office paper | office paper |
| 4 | 28 | 245 | 9 | softwood, pulpwood | wood pulp | office paper | office paper |

**Table S2.** Updated paper end-use ratios for HWP-C vR (rounded to the nearest hundredth).

| **Year** | **Newspaper** | **Coated paper** | **Corrugated Containers** | **Office paper** | **Other paper** |
| --- | --- | --- | --- | --- | --- |
| 1952-1960 | 0.31 | - | 0.32 | 0.15 | 0.23 |
| 1970 | 0.27 | - | 0.36 | 0.14 | 0.23 |
| 1980 | 0.24 | - | 0.37 | 0.16 | 0.22 |
| 1990 | 0.22 | 0.10 | 0.38 | 0.12 | 0.19 |
| 2000 | 0.20 | 0.10 | 0.39 | 0.11 | 0.20 |
| 2005 | 0.18 | 0.11 | 0.41 | 0.10 | 0.20 |
| 2010 | 0.16 | 0.09 | 0.46 | 0.10 | 0.18 |
| 2011 | 0.15 | 0.10 | 0.48 | 0.10 | 0.18 |
| 2012 | 0.14 | 0.09 | 0.49 | 0.09 | 0.18 |
| 2013 | 0.13 | 0.09 | 0.50 | 0.09 | 0.18 |
| 2014 | 0.13 | 0.09 | 0.51 | 0.09 | 0.19 |
| 2015 | 0.11 | 0.09 | 0.53 | 0.09 | 0.18 |
| 2016 | 0.11 | 0.09 | 0.53 | 0.09 | 0.19 |
| 2017 | 0.09 | 0.08 | 0.56 | 0.08 | 0.19 |
| 2018 | 0.09 | 0.08 | 0.57 | 0.08 | 0.19 |
